# Teaching self-efficacy and its effects on quality of bedside teaching: Findings from a multi-center survey

**DOI:** 10.30476/JAMP.2021.91264.1438

**Published:** 2022-04

**Authors:** ALI ASGHAR HAYAT, KARIM SHATERI, SEPIDEH KAMALIAN FARD, ELNAZ SABZI SHAHR BABAK, HATAM FARAJI DEHSORKHI, MOHAMMAD HASAN KESHAVARZI, KIMIYA KALANTARI, ALIREZA SHERAFAT, SEYED ABDOLLAH GHASEMTABAR

**Affiliations:** 1 Clinical Education Research Center, Shiraz University of Medical Sciences, Shiraz, Iran; 2 Department of Primary Education, Abdanan Center, Islamic Azad University, Abdanan, Iran; 3 Shiraz University of Medical Sciences, Shiraz, Iran; 4 State Management Training Center, Tehran, Iran; 5 Isfahan University of Medical Sciences, Isfahan, Iran; 6 School of Medicine, University of Central Lancashire, Preston, England; 7 Department of Educational Technology, Kharazmi University, Tehran, Iran

**Keywords:** Teaching, Self efficacy, Medical students

## Abstract

**Introduction:**

Evidence suggests that the performance of medical students is affected by the quality of teaching of clinical teachers, and the higher teachers’ teaching quality leads to better students’ clinical performance. Hence, the present research aimed to investigate the association between teaching self-efficacy and quality of bedside teaching among medical teachers.

**Methods:**

This is a cross-sectional study. To this end, 242 medical teachers and 830 medical students from 6 universities in different cities were selected using convenience sampling. The medical teachers filled out Physician Teaching Self-Efficacy Questionnaire (α=0.93), and medical students completed the quality of bedside teaching questionnaire (BST) (α=0.91). Confirmatory factor analysis (CFA), Pearson correlation coefficient, and multiple regression were used to analyze the collected data through SPSS 23 and Smart-PLS3 software.

**Results:**

The results of confirmatory factor analysis (CFA) demonstrated that all items and measurement models had adequate reliability and validity to enter the final analysis (α>0.7, CR>0.7 AND AVE>0.50). Furthermore, the results showed teaching self-efficacy (r=0.27, p<0.001) and its components including self-regulation (r=0.24, p<0.001), dyadic regulation (r=0.22, p<0.001), and triadic regulation (r=0.33, p<0.001) had a positive and significant relationship with quality of bedside teaching. Also, the results of multiple regression revealed that among the predictor variables, only the triadic regulation variable could predict the quality of bedside teaching of medical teachers (β=0.326, p<0.001).

**Conclusion:**

According to the findings, as the medical teachers’ teaching self-efficacy improves, they can provide high-quality teaching to students, which in turn will lead to better learning and, therefore better performance for medical students.

## Introduction

Many studies in medical education context have provided evidence that students’ performance is affected by clinical professors, and good professors may train students with better clinical performance and more clinical knowledge ( 1 - 3 ). Several studies have shown good teaching quality is among essential features of an excellent clinical teacher ( 4 ). High-quality patient care can only be expected if medical students have been provided with quality teaching during their studies in medical schools ( 5 , 6 ). In a non-medical context, researches showed that the quality of teaching was considered essential for students’ learning ( 7 , 8 ). Medical students’ development begins in an academic setting and continues into a clinical setting. Clinical teachers provide most teaching in this setting; thus, these doctors must be effective and good teachers ( 9 , 10 ). One of the mostly used and common educational strategies in the clinical setting is bedside teaching ( 11 , 12 ), which is an essential part of medical education and one of the best efficacious methods for learning communication and clinical skills ( 11 , 13 ). 

Bedside teaching is defined as teaching in the presence of a patient. Generally, it is thought that bedside teaching is applicable only to the hospital setting. However, bedside teaching skills apply to any situation where the teaching occurs in the presence of a patient, including an office setting and long-term care facility ( 14 ). 

High quality of bedside teaching lay the ground for the learning of professional behaviors ( 11 ). This delivers active learning in a real context; enhances the students' professional thinking, clinical reasoning and motivation, and skills; integrates problem-solving, communication, clinical, ethical, procedural, and decision-making skills; and increases the patients' understandings ( 12 , 13 , 15 - 19 ). Despite the importance and role of bedside teaching, studies show that its frequency and quality are declining ( 20 ). Some researchers have attributed this to low confidence and low self-efficacy ( 11 ). Psychological mechanisms are potentially influential factors in providing high-quality teaching, one of which is self-efficacy ( 21 , 22 ). Self-efficacy of teachers represents a job-specific individual trait ( 23 ) which can explain the differences in the methods of teaching and learning of students ( 24 , 25 ). Teachers' self-efficacy refers to the extent to which they feel they can favorably tackle conditions, situations, or tasks in the teaching profession (e.g., using new teaching methods, teaching difficult learners, resolving conflicts in social relations) ( 25 - 27 ). Most scholars in the field of teacher self-efficacy look at this construct as a major factor that leads to such positive educational consequences as effective classroom management ( 28 ), use of innovative teaching approaches ( 29 ), and setting of higher learning goals for students ( 30 ) or students motivation and achievement ( 31 , 32 ). 

Therefore, it can be concluded that the instructional behavior and outcomes of self-efficacious teachers in the classroom are different from their counterparts. The results of a meta-analysis showed a positive association between self-efficacy and job performance ( 33 , 34 ). Künsting et al. revealed that teacher self-efficacy remains a long-time and relatively stable forecaster of teaching quality ( 21 ). Holzberger et al., in their research, found that teacher self-efficacy is positively correlated to teaching quality ( 35 ). It can be mentioned that if physicians' teaching performance affects the students' learning progress, then teaching self-efficacy by physicians might play a critical role in forecasting the quality of their teaching.

Despite what was stated, up to now, few studies have been carried out on the effect of teacher self-efficacy on the quality of teaching in non-medical contexts ( 21 , 35 ).

**Figure 1:**
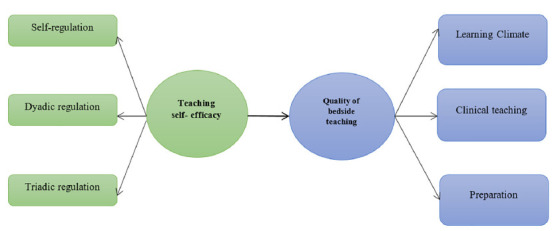
The conceptual model

Furthermore, the majority of the mentioned studies have examined the general self-efficacy of teachers ( 21 ), and less attention has been paid to teaching self-efficacy. Only in one study conducted in medical context, the influence of teaching self-efficacy on teaching quality has been evaluated, and its result was contradictory ( 22 ). Given the sensitivity and importance of medical students' learning, which obviously shapes their upcoming performance, researchers intend to investigate whether teachers' self-efficacy beliefs about their teaching can lead to more efficient delivery of academic content to students and thus lead to effective learning. Therefore, the present study aimed to investigate the relationship between teaching self-efficacy and its dimensions with the quality of bedside teaching and, in particular, examine whether teaching self-efficacy of medical teachers has a significant impact on their quality of bedside teaching. In the following part, the conceptual model of the research and the research hypotheses are presented (Figure 1).

## Methods

A cross-sectional study design was employed to carry out the current investigation in six universities of medical sciences. The data were collected during 11 months from April 3, 2019, to March 15, 2021. Participating universities that were selected using convenience sampling included all types, one, two, and three universities (Shiraz, Tehran, Isfahan, Kashan, Jahrom, Kerman). At each university, medical teachers who provided bedside teaching to students received a questionnaire composed of two parts: socio-demographic information section (age, gender, rank, etc.) and a scale to measure teaching self-efficacy. Totally, two-hundred and fifty-seven questionnaires of the 395 were turned back, revealing a response rate of 65%. Fifteen questionnaires were excluded from the ultimate analysis because they were not appropriately responded. Simultaneously, regarding students, 1,200 questionnaires were distributed, of which 892 were returned. In the initial screening, 62 questionnaires were discarded because they were not properly answered. Finally, 830 students of the same teachers answered a questionnaire rating various features of teaching quality regarding the specific BST lesson. Both faculty members and students were selected using convenience sampling.

In the present study, two valid and reliable questionnaires were used: 

### Teaching self-efficacy (TSE) 

A valid and reliable questionnaire called Physician Teaching Self-Efficacy Questionnaire (PTSQ) was applied to evaluate teaching self-efficacy (TSE) among medical teachers ( 36 ). This questionnaire contains 16 items based on a five-point Likert-scale that reflects medical teachers’ beliefs to deliver high-quality clinical teaching when fronting onto repeatedly happening important teaching positions like time pressures, patient selection, and related problems, allocating little time to lessons by teachers, disruptions of the lessons, or uninterested students ( 22 , 36 ). The validity and reliability of TSE were tested using confirmatory factor analysis (CFA) applying PLS software, and as indicated in Table 1, TSE and its components retained appropriate reliability and validity. 

**Table 1:** The results of confirmatory factor analysis

Variables	α	CR	AVE	Convergent Validity
Self-efficacy	0.93	0.94	0.62	Confirmed
Self-regulation	0.86	0.90	0.64	Confirmed
Dyadic regulation	0.84	0.89	0.63	Confirmed
Triadic regulation	0.83	0.88	0.59	Confirmed
Quality of bedside teaching	0.91	0.92	0.52	Confirmed
Learning climate	0.82	0.83	0.59	Confirmed
Clinical teaching	0.82	0.87	0.53	Confirmed
Preparation	0.78	0.85	0.54	Confirmed

Not: α (*Cronbach's* Alpha), CR (Composite Reliability), and AVE (Average Variance Extracted)

### Quality of bedside teaching

To assess the quality of bedside teaching (BST), Drilling et al.’s (2017) questionnaire was used ( 12 ). This measure has been developed to evaluate bedside teaching quality; it contains 18 items (Five-point Likert scale) and three components as follows: 1) learning climate, 2) clinical teaching, and 3) preparation. Drilling et al. reported good psychometric indices for this measure; these indices have also been approved in Iran by Jahromi et al. ( 37 ). Besides, to assess the reliability of the BST, we used both Cronbach's alpha and composite reliability (CR). Also, we used the AVE to test the validity of BST (Table 1).

To analyze the data, we firstly used Smart-PLS 3 to conduct confirmatory factor analysis to get the psychometrics of the scale in the new culture and new setting. Scholars suggest that adopted scales with sufficient empirical and theoretical evidence can be taken directly to CFA without running EFA beforehand ( 38 ). CFA is a more powerful method than relying on approaches like Cronbach’s alpha to validate a factor or scale reliability ( 39 ). Also, SPSS version 21 was applied to calculate standard deviation and mean as well as Pearson correlation coefficient and multiple regression at a significance level of 0.05.

### Ethical Considerations

Initially, the ethical approval of the current research was received through the Ethics Committee of Shiraz University of Medical Sciences (IR.SUMS.REC.1398.435); then, we obtained the participants’ written informed consent and asked the participants to complete the anonymous questionnaire voluntarily. We also assured them that their data would remain confidential and anonymous. 

## Results

A total of 257 medical teachers and 892 students completed and returned the questionnaires. Several questionnaires were considered invalid (no response on Average score or the same response for every item) and excluded from the final analysis process. Table 2 contains the details of the descriptive findings. As shown in Table 2, 51.7% of the medical teachers were male professors, and the other 43.4% were female professors. In addition, 81% of the participants were married. Also, in terms of rank, assistant professors and instructors had the highest and the lowest frequencies, respectively. According to Table 2, professors with 1 to 10 years of experience had the highest frequency, and professors with work experience of 21 years and above had the lowest frequency. Finally, as specified in Table 2, 35.9% of the medical students were male, and the other 57.22% were female. In addition, 74.33% of the students were single.

**Table 2:** Medical teachers and students’ demographic information

Variables		Medical teachers		Medical students	
Frequency	Percentage	Frequency	Percentage
Gender	Male	125	51.7	298	35.90
	Female	105	43.4	475	57.22
	Missing	12	5	57	6.86
Marital	Single	34	14	617	74.33
	Married	196	81	146	17.59
	Missing	12	5	67	8.07
Rank	Instructor	23	9.5	-	-
	Assistant	122	50.4	-	-
	Associate	37	15.3	-	-
	Professor	25	10.3	-	-
	Missing	35	14.5	-	-
Experience	1 to 10 years	117	48.3	-	-
	11 to 20 years	63	26	-	-
	21 years and older	33	13.6	-	-
	Missing	29	12	-	-
City	Tehran	36	14.9	75	9.03
	Isfahan	55	22.7	247	29.75
	Shiraz	39	16.1	137	16.5
	Kerman	32	13.2	119	14.33
	Kashan	38	15.7	118	14.21
	Jahrom	42	17.4	125	15.06

Moreover, as shown in Table 1, we used Cronbach's alpha and composite reliability (CR) to test the internal consistency of TSE, and all constructs retained values above the suggested standard (α≥ 0.7, CR≥ 0.7) (Table 1). Also, TSE and its components retained a suitable average variance extracted (AVE) (AVE≥ 0.5), varying from 0.59 to 0.64, which demonstrated the validity of constructs. 

In addition, as depicted in Table 1, BST and its components indicated appropriate composite reliability (CR≥ 0.7) and Cronbach's alpha (α≥ 0.7) which confirmed the internal consistency of the constructs. Finally, BST and its components maintained appropriate AVE (AVE≥ 0.5), varying from 0.52 to 0.59. Hence, the constructs’ validity and reliability were approved (Table 1). Given the confirmatory factor analysis findings, all questions submitted a loading of more than 0.7 on their related construct.

The results of confirmatory factor analysis showed that all questions submitted a loading more than 0.7 on their related construct varying from 0.71 to 0.84, which were significant at 0.05 level (t= 1.98). As a result, they retained the necessary prerequisites to enter the final analysis.

Then, to test the first hypothesis, we applied Pearson correlation coefficient to calculate the correlation between the research variables. The findings are presented in Table 3. The results indicated that teaching self-efficacy and quality of bedside teaching are positively and significantly correlated (r=0.27, p≤ 0.01). Also, the finding showed that self-regulation (r= 0.24, p≤ 0.01), dyadic regulation (r= 0.22, p≤ 0.01), and triadic regulation (r= 0.33, p≤ 0.01) were also positively correlated with the quality of bedside teaching. In addition, as presented in Table 4, teaching self-efficacy was also positively correlated with the quality of bedside teaching components (p≤ 0.01).

**Table 3:** The results of correlation matrix

Variable	1	2	3	4	5	6	7	8
1- Self-efficacy	1							
2- Self-regulation	0.87**	1						
3- Dyadic regulation	0.88**	0.62**	1					
4- Triadic regulation	0.89**	0.68**	0.71**	1				
5- Quality of bedside teaching	0.27**	0.24**	0.22**	0.33**	1			
6- Learning climate	0.11	0.06	0.09	0.18**	0.84**	1		
7- Clinical teaching	0.36**	0.32**	0.29**	0.38**	0.94**	0.70**	1	
8- Preparation	0.17**	0.15*	0.13*	0.20**	0.87**	0.65**	0.71**	1

*p < .05 **p < .01

**Table 4:** Results of multiple regression to identify the contribution of teaching self-efficacy components for predicting bedside teaching quality

Model	Unstandardized Coefficient		Standardized Coefficient	F	t	p
	B	Standard Error	Beta			
(Constant)	2.526	0.276		24.769	8.78	<0.001
Triadic regulation	0.348	0.126	0.326		4.98	<0.001
Self-regulation	0.030	0.123	0.029		0.236	
Dyadic regulation	-0.082	0.113	-0.087		-0.724	

Predictors: (constant), triadic regulation; Dependent variable: quality of bedside teaching

Considering the significance of the correlation coefficients between the research variables, we used multiple regression analysis to specify independent variables proportion (self-regulation, dyadic regulation, and triadic regulation) in predicting the dependent variable (quality of bedside teaching). Based on the findings, only the triadic regulation component (β= 0.326, P<0.001) could positively predict the quality of bedside teaching. In other words, triadic regulation explained 10% of quality of bedside teaching variance (R^2^=0.10).

## Discussion

This research aimed to explore the predictive role of physicians’ teaching self-efficacy in bedside teaching quality at five universities of medical sciences. As we hypothesized, physicians’ teaching self-efficacy exerted a significant and positive influence on the quality of bedside teaching. In line with prior research ( 21 , 35 , 40 ), teachers with more self-efficacy beliefs possessed a higher quality of teaching from the students' point of view, as indicated by the three dimensions of learning climate, clinical teaching, and preparation, whether teaching quality was rated by the medical students and teachers’ self-efficacy regarding to teaching was evaluated by themselves. The findings revealed that the more self-efficacy medical teachers possessed, the more likely their students were to perceive their teaching quality.

Many studies have revealed that teacher self-efficacy beliefs are among the most key factors in teacher’s competence ( 41 ) and fulfill an influential task in educational processes; that is, teachers who keep high efficacy beliefs have less stress, and more persistence, and are more engaged in non-formal learning activities ( 35 , 42 ). Therefore, it can be claimed that from a theoretical point of view, the level of teachers' performance can be affected by their self-efficacy ( 33 ). As supported by Bandura's social cognitive theory, it can be asserted that self-efficacy can indirectly affect motivation, and this effect can be explained through various cognition-directed behaviors and cognitive procedures. Beliefs attributed to self-efficacy motivate individuals in some ways. They specify the individuals' goals, the amount of their attempts, the degree of their perseverance in the face of difficulties, and their degree of resiliency in the face of failures ( 43 ). Therefore, as shown in some studies, these cognitions and behaviors affect the teachers' teaching performance ( 21 , 35 ). 

Especially, prior researches demonstrated that teachers’ beliefs related to their self-efficacy are connected to their instructional behaviors ( 40 ). In particular, teachers with more self-efficacy can manage their class more effectively than those with less self-efficacy ( 44 ), employ innovative teaching methods ( 45 ), show further persistence in problematic situations ( 43 ), deal better with stress, exhibit larger levels of organization and planning, demonstrate further openness facing latest teaching methods and strategies which better suit the students’ needs ( 46 ). Research has repeatedly shown that teachers’ self-efficacy impacts their teaching effectiveness as well as their effective efforts and behaviors in the classroom ( 47 ). 

Some studies have shown that teachers with superior self-efficacy are more willing to employ novel teaching methods, to better organize and plan their classrooms, show higher instructional quality ( 21 , 35 ), utilize further distinctive instruction and constructivism ( 48 ), develop challenging lessons ( 49 ), employ instructional procedures to stimulate the students’ independence, engage the students in their lessons ( 44 ), and are more eager to teach and more satisfied with teaching than others ( 46 , 50 ). To put it concisely, self-efficacy feelings influence teaching and attitudes toward the educational process, which in turn improve the teaching and learning quality. Additionally, some researchers have supposed that high-self-efficacy instructors positively enhance the classroom learning environments accompanied by high-quality curriculum planning and purposeful teaching ( 51 ), so that all these are prerequisites of effective and quality teaching and students’ learning ( 52 ).

The current study maintained several strengths, one of which represents our focus on the quality of bedside teaching (BST), which covers a critical component of clinical education. This study was also performed at the level of several universities and in the form of multi-center, which increases the validity and the generalizability of the findings. On the other hand, rating the professors' quality of bedside teaching (BST) by several students reduces the possibility of evaluation biases. Another strength of this research is the use of valid and special medical context questionnaires that ensure the validity of the results.

### Limitations and applications

The mere application of student ratings can include biases, so for future studies, it is recommended that other sources of teaching quality evaluation, including clinical observation, video-based classroom analysis, or a peer evaluation should be utilized. Also, assessing the students' learning achievement using tests may provide a more valid criterion to evaluate the teaching quality than the students' assessment. In this study, only the BST questionnaire was used, so it is suggested that future researchers should employ other types of lessons or a combination of them to assess the effect of self-efficacy on the teaching quality.

According to the findings of the current research, it can be stated that teaching quality might be increased by training the teachers to recognize the students’ competencies and also by increasing the physicians’ teaching self-efficacy. In this regard, Bandura has previously declared pleasant imaginary experiences, mastery experiences, oral persuasion, and subjective explanation of physical and emotional states throughout an action represent the primary sources of self-efficacy, and this claim has been supported in various studies ( 13 , 14 ). Therefore, to effectively improve the physicians’ teaching self-efficacy, these principles should be considered in teacher training programs. Since the association between self-efficacy and educational processes and behaviors is a reciprocal one, holding professional development courses in teaching methods can deepen their teaching self-efficacy feelings, which in turn can improve the quality of their teaching. It is also suggested that capacity-building workshops and programs should be held to increase the professors’ self-efficacy. For example, setting up a counseling center to support teachers overcome their teaching problems can expand their sense of competence and, consequently, their teaching self-efficacy.

## Acknowledgement

We thank the Research Deputy of Shiraz University of Medical Sciences (SUMS) for funding this research (16490). Also, we wish to thank all the medical students who participated in this study. 

**Conflict of Interest:** None Declared.
